# Understanding Patient Experiences of Vulvodynia Through Reddit: Qualitative Analysis

**DOI:** 10.2196/63072

**Published:** 2025-03-06

**Authors:** Aurora J Grutman, Sara Perelmuter, Abigail Perez, Janine Meurer, Monica Contractor, Eva Mathews, Katie Shearer, Lindsey A Burnett, Maria Uloko

**Affiliations:** 1 Johns Hopkins School of Medicine, Baltimore, MD, United States; 2 Weill Cornell Medical College, New York, NY, United States; 3 NYIT College of Osteopathic Medicine, Glen Head, NY, United States; 4 Jacobs School of Medicine and Biomedical Sciences at the University at Buffalo, Buffalo, NY, United States; 5 Loyola University Chicago Stritch School of Medicine, Chicago, IL, United States; 6 Department of Family Medicine, Community Memorial Healthcare, Ventura, CA, United States; 7 University of Tennessee Health Science Center, Memphis, TN, United States; 8 Department of Obstetrics, Gynecology and Reproductive Sciences, University of California, San Diego, CA, United States; 9 Department of Urology, University of California, San Diego, CA, United States

**Keywords:** sexual health, health literacy, vulvodynia, vestibulodynia, pelvic pain, Reddit

## Abstract

**Background:**

Vulvodynia is a chronic vulvar pain condition affecting up to 25% of the US population. However, diagnosis and effective treatment remain elusive. Many individuals with vulvodynia face stigma and medical uncertainty, leading them to seek information and web-based support. Reddit is a popular social media platform where patients share health concerns and experiences. The anonymity and accessibility of this platform make it a valuable source of real-world patient perspectives that are often overlooked in clinical settings.

**Objective:**

This study evaluated Reddit content related to vulvodynia to explore how individuals with vulvodynia describe their symptoms, treatments, and personal experiences.

**Methods:**

The subreddits “r/vulvodynia” and “r/vestibulodynia” were selected for analysis in May 2023. Threads were sorted from the most popular to least popular, with “popularity” measured by upvotes. Opening threads from the top 70 posts in each subreddit were extracted and analyzed using inductive qualitative analysis to identify themes and sentiment analysis to evaluate attitudes.

**Results:**

In May 2023, the “r/vulvodynia” and “r/vestibulodynia” subreddits had a total of 7930 members (7245 and 685 members, respectively). Out of 140 analyzed threads, 77 (55%) contained negative attitudes. A total of 50 (35.7%) threads were seeking information or advice and 90 (64.3%) included some form of peer support. Inductive thematic analysis identified 6 core themes: symptoms (n=86, 61.4%), treatments (n=83, 59.3%), sexuality (n=47, 33.6%), erasure or disbelief (n=38, 27.1%), representation or media (n=17, 12.1%), and humor (n=15, 10.7%). Threads that discussed treatments (48/83, 57.8%), sexual experiences (25/47, 53.2%), and representation (14/17, 82.4%) had the highest proportions of positive attitudes, while threads that touched on erasure (21/38, 55.3%), symptoms (51/86, 59.3%), and humor (12/15, 80%), had the highest proportion of negative attitudes. A multivariable logistic regression of valence on the themes revealed that posts referring to treatments (odds ratio 12.5, 95% CI 3.7-42.2; *P*<.001) or representation (odds ratio 21.2, 95% CI 4.2-106.0; *P*<.001) were associated with significantly increased odds of positive valence. Furthermore, it was noted that 3 of the 5 most frequently discussed treatments aligned with clinical guidelines from the American College of Obstetricians and Gynecologists, American Urological Association, and International Society for the Study of Vulvovaginal Disease. Despite this alignment, threads frequently mentioned alternative remedies and frustration with medical professionals related to diagnostic delays and perceived lack of understanding.

**Conclusions:**

This is the first study of Reddit discussions about vulvodynia. Findings suggest a gap between patient experiences and provider understanding, underscoring the need for improved patient education and greater clinician awareness of psychosocial factors in vulvodynia care. While limited by its sample size and lack of demographic data, this study highlights how web-based communities can help identify ways health care providers can better meet patient needs and how patients mutually support each other.

## Introduction

Vulvodynia is defined as vulvar pain lasting for at least 3 months without an identifiable cause. Vulvodynia is characterized by the location of pain (eg, localized or generalized), triggers (provoked, spontaneous, or mixed), onset (eg, primary or secondary), and temporal nature (eg, intermittent, constant, or delayed) [1,2]. The most common subtype of vulvodynia is vestibulodynia, which is pain isolated to the vulvar vestibule [2].

Vulvodynia is prevalent, affecting up to 1 in 4 women in the United States [3,4]. However, the majority of people living with vulvar pain remain undiagnosed and inadequately treated. It is reported that nearly 40% of people with chronic vulvar pain do not seek treatment, and of those that do, 60% consult at least 3 physicians before receiving a diagnosis, if they receive one at all [3]. Factors contributing to this gap in care may include a lack of knowledge on the part of medical providers, inadequate medical education related to vulvar anatomy and physiology, longstanding dismissal of female pain, and stigma surrounding female reproductive organs and sexuality [5-7].

Since the advent of the internet, individuals have sought web-based medical information, often before consulting health care professionals [8,9]. For individuals with understudied health conditions, digital health forums can be essential sources of information and peer support [10]. Moreover, web-based platforms may allow individuals with chronic diseases to connect with one another and build a social identity that extends beyond the disease itself [11].

Reddit, a popular website with 1.5 billion registered users and over 52 million daily users is a notable platform for exchange and anonymized information-sharing [12]. By design, Reddit facilitates open discussion across various topics, allowing for global information exchange that is not as readily facilitated on other platforms such as Instagram or Facebook. Reddit is organized into various subreddits, which are discussion-based communities devoted to an identified topic or theme. Within a typical subreddit, a user makes a post expressing an opinion or sharing information. Other users can evaluate that post by “upvoting” or “downvoting” it. Users can reply to the initial post or others who have replied to the post. Together, the post and subsequent comments are known as a “thread.”

The anonymous nature of Reddit may be beneficial because it lends itself to open and often vulnerable exchanges. Prior studies have explored Reddit content related to substance use, chronic pain, sexual dysfunction, and mental health, providing key insights into the lived experiences of those who may feel shame [13-17]. Despite relatively extensive investigation of these topics with varied analytic techniques, there is little research on Reddit content related to female sexual dysfunction. Existing studies primarily focus on reproductive conditions, changes in libido, or abortion [18-22]. To date, there has been no study examining how Reddit may be used by patients to obtain or share information about vulvodynia.

While population-level data on patient experiences of vulvodynia exists, patient-centered studies that capture the experiences of individuals living with vulvodynia are rare [23-25]. This study therefore seeks to qualitatively assess patient experiences of vulvodynia as discussed on Reddit, amplifying person-centered perspectives and gauging gaps in medical care for those affected by vulvodynia. By capturing these narratives, this study highlights the importance of understanding patient experiences beyond the clinical setting, which can inform more empathetic and effective health care strategies.

## Methods

### Subreddit Selection

To evaluate Reddit forum content related to vulvodynia, this study used a cross-sectional design, analyzing publicly available data from “r/vulvodynia” and “r/vestibulodynia,” in May 2023 [26,27]. The “r/vestibulodynia” subreddit was included to provide a more comprehensive understanding of patient experiences, as vestibulodynia is a specific form of vulvodynia.

For each subreddit, threads were sorted from the most popular to the least popular. A thread becomes popular based on the number of upvotes, comments, and overall engagement it receives from others in the Reddit community. We collected and analyzed the most popular 70 threads from each subreddit, as they were deemed most representative of key topics in the community. Comments from other users on each thread were excluded from the analysis.

### Thematic and Sentiment Analysis

Quantitative and qualitative data, including the number of upvotes and comments, the post title, and a brief description of the post, were collected by accessing the Reddit website directly and navigating subreddits directly through the site. Data were collected using Google Chrome (version 113) and Safari (version 16.5) web browsers under default settings (cache and cookies enabled, no use of privacy or incognito mode) to simulate a typical user experience. The data were directly downloaded and preserved in an Excel (version 16.82; Microsoft Corp) spreadsheet on May 7, 2023, at approximately 6 PM EST. Four team members (SP, EM, KS, and AP), located in New York, Ventura, Memphis, and Glen Head, served as coders. Extracted data were coded between May 7, 2023, and May 15, 2023. These coders independently assessed all 140 threads, using a thematic analysis approach. All posts were manually annotated, and the themes were derived through iterative review and comparison. Codes were revised as necessary based on commonly identified themes, following established qualitative analysis procedures and an inductive approach in which the analysis is guided by the data itself, allowing for themes to emerge organically [28-30]. Each thread was assigned 1 or multiple themes. Threads were also evaluated for tonal expression. We defined “positive” attitudes to be any expression of optimism, relief, or joy, as well as references to “cures,” and observational or light humor. “Negative” attitudes were defined as any expression of frustration, despair, fear, or isolation, as well as mentions of pain, pessimism, exhaustion, or unresolved symptoms, and dark humor with elements of bitterness or the macabre or morbid.

Any discrepancies in themes and attitudes based on individual coding were identified by AJG who was not involved in coding. All instances of coding discrepancy were resolved by group consensus, a practice used in qualitative research to constructively arrive at a consistent understanding of the data [31,32]. For example, in cases where coding defined the post as negative because of a user’s description of vulvodynia as “terrible,” but also noted that the post was “positive” because it used “humor” together, the coding team engaged in a discussion of whether the post should be coded as “negative” due to the word “terrible,” or as “positive” because the presence of humor suggested a more complex emotional response, such as a coping mechanism. Examples of representative threads by theme and valence are provided in Multimedia Appendix 1.

A multivariable logistic regression of valence on the 6 identified themes was conducted using STATA/BE (version 18.0; StataCorp LLC). Each post was assigned a binary code for positive or negative valence and the presence or absence of a particular theme. A 2-sided significance level was defined at ɑ=.05.

Any mention of treatments in the subreddit was recorded and compared with guidelines from the American College of Obstetricians and Gynecologists (ACOG), American Urological Association (AUA), and International Society for the Study of Vulvovaginal Disease (ISSVD), as they are viewed as the primary sources of information about managing vulvar pain.

### Ethical Considerations

The study was deemed exempt by the Institutional Review Board of the University of California San Diego and Johns Hopkins Institutional Review Board due to its observational nature and analysis of public web-based content. The original data were collected in compliance with Reddit’s public content policy, which informs users that researchers can access Reddit’s public content for research purposes. All Reddit usernames and any potentially identifiable information were deidentified to protect user privacy. Furthermore, no direct user interactions or private messages were included in the analysis. Only publicly accessible forum posts were analyzed, and efforts were made to ensure that the data could not be traced back to individual users through reverse searchability. In consideration of the potential ethical concerns related to social media–based research, the authors acknowledge the need to engage in ongoing academic debates regarding internet research ethics. While Reddit users agree to the public visibility of their posts, the authors recognize that these ethical discussions, such as those put forth by the Association of Internet Researchers, underscore the need to balance public data use with user privacy in research contexts.

## Results

At the time of analysis, the “r/vulvodynia” and “r/vestibulodynia” subreddits had a combined total of 7930 members (7245 and 685 members, respectively). A total of 140 posts were analyzed; these posts received an aggregate of 4166 upvotes. Out of all 140 analyzed threads, 50 (35.7%) were deemed to be seeking information or advice and 90 (64.3%) were deemed to involve peer support discussions of personal experiences related to vulvodynia.

Six core themes emerged from the qualitative analysis: (1) Reddit users’ subjective sense of being disbelieved about symptoms or erasure more generally; (2) difficulty managing symptoms; (3) the condition’s impact on sexuality and sexual experiences; (4) representation or media; (5) humor as a coping technique or a response to the condition; and (6) treatments sought or tried.

Out of the 140 threads, the most frequently observed themes were symptoms (n=86, 61.4%) and treatments (n=83, 59.3%), followed by sexuality (n=47, 33.6%), erasure or disbelief (n=38, 27.1%), representation or media (n=17, 12.1%), and humor (n=15, 10.7%). Of all 140 analyzed threads, 45% (n=63) of threads were coded as reflecting positive attitudes, and 55% (n=77) of threads were coded as reflecting negative attitudes. The core themes of treatments (48/83, 57.8%), sexual experiences (25/47, 53.2%), and representation (14/17, 82.4%) had the highest proportions of positive attitudes in analyzed threads. The themes of humor (12/15, 80%), erasure or disbelief (21/38, 55.3%), and symptoms (51/86, 59.3%) had the highest proportions of negative attitude threads. Fleiss κ for coded valence was 0.54. The distribution of positive and negative attitudes across themes is illustrated in Table 1.

Results from the multivariable logistic regression revealed that only treatments (odds ratio [OR] 12.5, 95% CI 3.7-42.2; *P*<.001) and representation (OR 21.2, 95% CI 4.2-106.0; *P*<.001) were associated with significantly increased odds of positive valence. Nonsignificant associations were found for themes erasure (OR 1.25, 95% CI 0.5-3.0; *P=*.60), symptoms (OR 0.47, 95% CI 0.2-1.2; *P*=.11), and sexuality (OR 2.2, 95% CI 0.9-5.1; *P*=.07).

There were 119 instances of treatment discussions across the 140 analyzed threads. The most commonly mentioned treatments included topical medications (n=22, 18.5%), physical therapy (n=22, 18.5%), surgery (n=16, 13.4%), dilators (n=14, 11.8%), and stopping oral contraceptive pills (n=11, 9.2%). Three of the 5 most discussed treatments—physical therapy, topical medications, and surgery—aligned with clinical guidelines from ACOG, AUA, and ISSVD.

**Table 1.** Positive and negative attitudes by theme.

Theme	Positive	Negative	Total
Erasure or disbelief	17	21	38
Symptoms	35	51	86
Treatments	48	35	83
Sexuality	25	22	47
Representation or media	14	3	17
Humor	3	12	15

## Discussion

### Principal Findings

This is the first study to analyze Reddit posts about vulvodynia. On Reddit, individuals with vulvodynia shared personal experiences, provided advice, and found communal support. From the qualitative and sentiment analyses, 6 core themes with unique valence distributions were identified, providing insight into the experiences, priorities, and needs of individuals living with vulvodynia.

### Reflections on Erasure and Being Disbelieved

A published study exploring the experience of women with vulvodynia in the United Kingdom found that health care professionals often dismiss patients’ expressions of concern or physicians lack knowledge about the condition [33]. The substantial percentage of posts mentioning not being taken seriously by a health care provider, which was coded as “erasure and disbelief” indicates health care’s inadequate support for patients with vulvodynia, which may explain the prevalence of negative attitude posts. Discussions of erasure and being disbelieved were present in many of the opening threads, and many users described needing to increase self-advocacy in medical settings. Such reports highlight the persistent marginalization and sense of being disbelieved during health care interactions, thereby necessitating substantial self-advocacy. Reddit users shared their disappointment with providers’ behavior, attitudes, and expertise: one user shared that her doctor bluntly asked if the patient had tried lubricant, revealing a gap in understanding and empathy about vulvodynia’s etiology and treatment. In aggregate, the prevalence of posts mentioning erasure and being disbelieved underscores the critical need for improved medical education and patient-centered care, 2 weaknesses of health care professionals at all levels of training [5,6,34-36].

### Physical Symptoms and the Impact on Daily Functioning

Symptoms were the most prevalent theme, and many posts emphasized the wide-ranging impact of symptoms on overall health. Symptom-related posts predominantly had a negative attitude, reflecting the disruptive nature of physical discomfort in all facets of daily life. It is essential to acknowledge, however, that participants posting in these threads may not all have a formal diagnosis of vulvodynia. It is impossible to verify the truth of the contents of any of the posts. Despite this limitation, however, there were notable parallels in the dataset between user-reported symptoms and clinical diagnostic criteria for vulvodynia. Currently, there is no exclusive classification for vulvodynia; rather, a diagnosis is characterized by the description of pain [1,2,16].

Pain was the most discussed symptom, underscoring the debilitating and all-consuming nature of vulvar pain [37]. Reddit users described experiences of burning pain, pain with tampon insertion, pain during sexual intercourse, and irritation from clothing. Some symptoms mentioned, such as swollen tissue associated with tampon insertion, pain with urination, and pelvic floor tightness, do not align with established diagnostic criteria, suggesting current diagnostic tools may not capture the full range of experiences of individuals living with vulvodynia [2].

Vulvodynia can interfere with day-to-day functioning; one user noted vulvar pain made it difficult to ride a bike. Others found it challenging to stay active due to pain. Difficulties related to vulvodynia extended beyond physical discomfort; one user described how finding comfortable and wearable underwear became an unexpected source of financial stress. The heterogeneity of pain associated with vulvodynia suggests that further research is needed to better understand its etiology and develop more effective treatment strategies.

In addition to pain, the subreddit posts included expressions of anger, frustration, anxiety, depression, and even trauma, highlighting the connection between mental health and chronic pain. Although few studies have investigated mental health outcomes in individuals with vulvodynia, current evidence suggests that vulvodynia symptoms contribute to worse quality of life and many individuals living with vulvodynia have comorbid anxiety or depression [38-40]. While further research is needed, comprehensive care for vulvodynia should consider both physical and mental health to improve patient well-being.

### Treatment Approaches: Navigating Options and Uncertainty

Treatment-related discussions highlight the range of difficulties individuals face in managing chronic health conditions. Participants exchanged information about various treatment modalities, sharing insights into effectiveness, side effects, and accessibility. Of posts discussing treatments, the higher proportion of positive opening threads suggests that individuals in this Reddit community often shared experiences of treatment that were effective. Three of the 5 most mentioned treatments in the Reddit threads—physical therapy, topical medications, and surgery—aligned with guidelines from ACOG, AUA, and ISSVD. To be sure, not all discussions of these treatments were positive. However, these discussions indicate that users in this web-based forum are aware of and discuss clinically recommended treatments.

Physical therapy and vaginal creams were the top 2 treatment modalities discussed. While physical therapy is widely recognized as an effective approach for vulvodynia, vaginal creams such as baclofen and amitriptyline, though effective, are still considered novel remedies [41-43]. Surgery and discontinuing oral contraceptives were also commonly discussed. Surgery is considered for cases where conservative methods fail [1,44]. Procedures such as vestibulectomy or neuromodulation aim to alleviate pain by removing affected tissue or modifying nerve signals. Although controversial, the AUA and ACOG recommend discontinuing hormonal contraceptive treatments, as these may worsen symptoms. The literature on this topic is divided, however. Some researchers argue that long-term oral contraceptive pills may contribute to vestibulitis, while others provide evidence that refutes this connection [45,46].

Another notable challenge discussed by Reddit users is the wide variation in rates of treatment success, an observation that is well-documented in the literature [2,3,37]. Success rates for medical interventions are reported to range from 13% to 67% [47]. Note that the AUA, ACOG, and ISSVD provide slightly differing guidelines for treating vulvar pain. This may complicate care for providers already navigating serious time constraints in health care. In light of Reddit users’ self-reported challenges in obtaining successful treatment for vulvodynia, harmonizing treatment guidelines would likely benefit clinicians and patients alike.

### Sexuality and Relationships: Coping With Intimacy Challenges

It is not surprising that sexual experiences also emerged as a prevalent theme in these subreddit threads, given that vulvodynia directly affects individuals’ intimate lives and sexual health [48]. One user shared that vulvar pain complicated their interest in sexual intimacy, demonstrating how the connection between experiences of pain, desire, pleasure, and sexual experiences may be altered by vulvodynia. Some users detailed the frustrations and challenges of finding an understanding partner, while others shared success stories of supportive and accommodating partners. Further research is needed to understand how vulvodynia impacts relationships and sexuality. In subsequent studies, qualitative interviewing would be one way to center the voices of individuals with vulvodynia.

### Media Representation and Visibility

The low percentage of posts discussing representation and media highlights the invisibility of vulvodynia to the public. The prevalence of positivity in such posts underscores the urgent need for increased awareness, which can be transformative for an individual’s sense of self and confidence. One user shared that representation in media made them feel less isolated in their experience. In this way, media may represent an unexpectedly positive domain in which individuals with vulvodynia can find support and recognition of their experience. Health care providers should be aware of the power of representation to positively impact individuals with vulvodynia who may feel overlooked by the medical system. For others, media can be a reminder of the difficulties associated with pain, sexuality, and daily functioning. Overall, representation was associated with significantly increased odds of positive valence, illustrating the value and importance of representation for individuals with vulvodynia.

### Humor as a Coping Strategy

Humor is well-recognized as an adaptive tool for coping with stressful situations. For individuals with chronic pain, in particular, humor has been shown to reduce pain intensity and improve quality of life [49]. Explicit humor therapy, in which individuals engage with materials they find entertaining, is associated with decreased pain and feelings of loneliness [50]. In this way, humor represents a nonpharmacological approach for addressing and even ameliorating pain. Humorous interpersonal interactions have also been noted as a way for individuals with chronic pain to engage with one another and even improve clinical outcomes [51]. Members of the vulvodynia community on Reddit creatively reframed their experiences through memes and conversational threads. Users generated memes and made jokes about symptoms and interactions with doctors; in this way, the separation of body and mind may be a method of relief. Humor therefore represents a unique approach for managing experiences of vulvodynia, and it is one means by which members of the Reddit community express themselves and connect with others.

### Limitations

A notable limitation of this study is the lack of access to user demographics due to the anonymous nature of Reddit. As a result, we were unable to interpret the possible effects of factors including race, age, health literacy, socioeconomic status, location, transportation, and access to health care which may have impacted the experiences mentioned by each user. Although we cannot determine whether any user had an official diagnosis or indeed met diagnostic criteria for vulvodynia, the Reddit contributors were driven to the platform for specific reasons. Furthermore, as a cross-sectional study, these results are only representative of the time in which data were collected. Results are not generalizable and should be understood as a snapshot of what anonymous Reddit users reported about vulvodynia.

### Conclusions

This study aimed to better understand patient experiences of vulvodynia by analyzing web-based discussions on Reddit. Findings highlight that Reddit serves as a vital platform for sharing personal experiences, accessing peer-to-peer support, and seeking health care–related information. These web-based discussions provide valuable anecdotal evidence underscoring a need for health care providers to be trained on the management of vulvodynia, guided by consensus from professional associations. Such training would help ensure patients receive accurate diagnoses and effective care. By prioritizing and centering the patient perspective, health care providers can gain a deeper understanding of the multifaceted challenges faced by individuals living with vulvodynia. This study contributes to existing literature by offering insights directly from those affected by vulvodynia or who are experiencing vulvodynia-like symptoms.

## Supplementary material

10.2196/63072 Multimedia Appendix 1 Example paraphrased threads by theme and valence.

## Abbreviations

ACOG: American College of Obstetricians and Gynecologists
AUA: American Urological Association
ISSVD: International Society for the Study of Vulvovaginal Disease
OR: odds ratio
