# Supplementary figures and images for: Understanding Patient Experiences of Vulvodynia Through Reddit: Qualitative Analysis
Source: JMIR Infodemiology. 2025 Mar 6;5:e63072. doi: 10.2196/63072 (PMC11906116; doi:10.2196/63072)

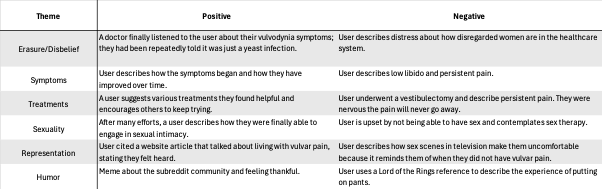

Supplement: Multimedia Appendix 1 [file infodemiology-v5-e63072-s001.png]
